# The association of autoantibodies expression, Th1/Th2 cytokines balance and IFNG polymorphism with histological phenotype of lupusnephritis

**DOI:** 10.1186/ar3681

**Published:** 2012-02-09

**Authors:** Kusworini Handono, Atma Gunawan, Singgih Wahono, Rulli Rosandi, Wivina, Natalina Mallo, Handono Kalim

**Affiliations:** 1 Clinical Pathology Department, Medical Faculty Brawijaya University/Dr Saiful Anwar Hospital Malang, Indonesia; 2 Internal Medicine Department, Medical Faculty Brawijaya University/Dr Saiful Anwar Hospital Malang, Indonesia

## Background

Racial differences were observed in clinical, serologic and histologic presentation of lupus nephritis (LN)[1]. It has been suggested that Th1/Th2 cytokines balance and IFNG polymorphism play important role in the development of different pathologic pattern of lupus nephritis (LN )[2-4]. The objective of our study is to determine the association between autoantibodies expression, Th1/Th2 cytokines balance and IFNG polymorphisms with pathologic class of LN in Javanese patients.

## Patients and methods

We studied 60 female patients with LN (ARA criteria,1992), and 20 healthy individual as control. Histopathologic classification was based on WHO criteria (1995). Anti ds-DNA, anti RO, anti nRNP and anti Sm autoantibodies were assayed by ELISA.IFNg-IL-4 balance were used to assess Th1/Th2 cytokines balance, IFNγ and IL4 serum levels assayed by ELISA. Microsatelitepolymorphisms within the first intron of the IFNG gene on chromosome 12q24.1 was performed by DNA sequencing. The association of histopathologic phenotype of LN with Th1/Th2 balance (IFNγ/IL4),and autoantibodies expression were analysed by Chi-square and Student T test with p < 0.05 is significant. The IFNG allele difference between LN classes were analysed by Chi-square. The risk of LN in patients with certain IFNG allele was calculated using Odds Ratio.

## Results

Our study showed that the frequency of anti Ro, and anti nRNP antibodies in patients with LN WHO class III, IV and V LN weresignificantly higher compared with patients with class I and II LN. There is no autoantibodies expression differences between class III, IV and clas V LN. The IFNγ/IL4 ratio in patients with classIII and IV LN was significantly higher than patients with class I,II and class V LN (2.30 ± 0.89 vs 0.94 ± 0.24 and 0.69 ± 0.30, p = 0.000), but the serum level of IL4 in patient with WHO class III and IV was significantly lower than class V (77.72 ± 40.28 pg/ml vs 145.68 ± 71.21 pg/ml. p = 0.014). The result showed that the activity of Th1 immune response tent to be higher in patient with WHO class III and IV LN. The frequency of IFNG 112 allele were higher in patients with SLE compared with healthy controls (55% vs 25%, p = 0.0471) and the risk to have LN class V in patients with IFNG 112 was 6 times higher compared with patients without these allele (OR 6.1, CI 95% 3.1- 12.4, p = 0.0427).

## Conclusion

The results showed different underlying mechanism of inflammation in different pathologic class of LN.
